# Ranking routes in semiconductor wafer fabs

**DOI:** 10.1038/s41598-023-39187-2

**Published:** 2023-08-15

**Authors:** Shreya Gupta, John J. Hasenbein, Byeongdong Kim

**Affiliations:** Graduate Program in Operations Research and Industrial Engineering, Department of Mechanical Engineering, University of Texas at Austin, Austin, TX 78712 USA

**Keywords:** Applied mathematics, Computational science, Scientific data, Software, Statistics

## Abstract

We develop a method to estimate the quality of processing routes in a wafer fabrication process. Ranking such routes can be useful for identifying the “best” and “worst” routes when making adjustments to recipes. Route categorization is also useful in developing efficient scheduling algorithms. In particular, we propose a method for ranking routes based on count-based metrics such as the number of defects on a wafer. We start with a statistical model to produce a “local” ranking of a tool and then build a “global” ranking via a heuristic procedure. Creating a fully statistical procedure for ranking routes in semiconductor fabrication plants is virtually impossible, given the number of possible routes and the limited data available. Nonetheless, our discussions with working engineers indicate that even approximate rankings are useful for making better operational decisions.

## Introduction

In this paper, we develop a method to estimate the quality of processing routes in a manufacturing process. This work was inspired by the situation in a typical semiconductor wafer fab, but the method could be used in any sector. However, some of our terminology and focus is driven by the application area we have in mind.

A semiconductor manufacturing facility is know as a *fab*. Typically a semiconductor manufacturing process constitutes semiconductor wafers (the entities being manufactured) moving through a sequence of *tools* (or equipment or machines) in a pre-specified order such that they can be appropriately processed by each tool. This pre-specified order of tools is referred to in the semi-conductor manufacturing industry as a *route*. Each tool in the route also has a pre-specified setting to get wafers of a certain quality. Together the route and the pre-specified setting for each tool in that route is referred to as a *recipe*.

In a semiconductor fab, there are typically several tools that can be chosen to complete a given step in the production process. A recipe, as described earlier, consists of a specific ordering of manufacturing steps plus the tool settings at each step. Ranking such routes can be useful for various reasons. First, identification of the “best” and “worst” routes is helpful in recipe probing. Specifically, when adjustments are made to tool settings, it is useful to identify the best and worst routes in the existing process as these are likely to provide good bounds on performance for the adjusted recipe. Second, route categorization may be used to aid efficient scheduling. For example, the route ranking can be used as one factor in dispatching jobs as they progress through the manufacturing process.

We develop a method for ranking routes for count-based metrics, in which the metric takes values that are non-negative integers and for which lower values are better. In particular, 0 is the best possible value of the metric. The computational example considered in this paper relates to defect counts on a wafer.

In general, our method starts by developing a “local” ranking of a tool and then builds a “global” ranking via a heuristic procedure. Note that it is not always possible to rank the tools directly from, say, defect data, because this data is often not collected until a product has undergone several processing steps. Hence, we must estimate the correlation between the defects and the tool choices. Similarly, creating a a detailed statistical procedure for ranking routes in semiconductor is virtually impossible, given the number of possible routes and the limited data available. Nonetheless, our discussions with working engineers indicate that even an approximate ranking is useful for making better operational decisions in the fab.

The rest of the paper is organized as follows. In “Literature review”, we briefly review the previous related work. In “Count-based route ranking”, we propose two ranking algorithms for count data: count regression based ranking and binary probability based ranking. In “Computational examples”, we illustrate the algorithms using some computational examples and compare the results of the two ranking algorithms. Finally, we wrap up the paper in “Conclusion” with suggestions on when to use one ranking algorithm versus the other.

## Literature review

There seems to be relatively little previous work on ranking routes in manufacturing. Chang et al.^1^ use the analytic hierarchy process (AHP) to rank three cutting tools based on precision. They further analyze how sensitive this ranking is to the weights of the criteria selected by the decision maker. Similarly, Chang et al.^2^ build a modified fuzzy AHP (FAHP) to rank tools based on the weights of selected criteria and analyze the sensitivity of these priorities to the criteria. Chang et al.^3^ examine three diamond cutting tools using the analytic network process (ANP), a generalization of AHP. They rank these tools in increasing order of the time required for tool examination and monitoring. The objective of the three aforementioned papers is to find the machine with highest precision that increases yield and reduces manufacturing cost. They do so by attempting to identify the characteristics and criteria affecting manufacturing quality. A strength of their analysis is that they use AHP, which can combine qualitative and quantitative factors in the ranking.

A similar problem is tackled in Rao and Patel^4^ which addresses ranking alternative manufacturing tool options using the preference ranking organisation method for enrichment evaluations (PROMETHEE) integrated with AHP and fuzzy logic. The paper proposes the use of an improved PROMTHEE method that uses AHP to calculate the relative importance of different criteria. Thus, these weights are based on the decision makers’ preferences. Further, PROMTHEE also involves the use of a preference function for the decision maker. A benefit of using PROMTHEE is that it allows as many qualitative and quantitative criteria as desired, and computationally feasible. Furthermore, it takes the relative importance of these criteria into consideration. Chakraborty^5^ ranks advanced manufacturing systems using data envelopment analysis to identify a homogeneous group of “good” systems and then uses their technical differences to further distinguish them. It also weighs these technical attributes based on their importance and then proposes a final rank. This methodology provides a complete ranking of the alternatives from the best to the worst and also takes user preference into account.

Chien et al.^6^ use the Kruskal–Wallis and multiple comparison tests to differentiate and recognize problematic and normal tools based on yield-loss. They then perform ANOVA and regression analysis on the extracted yield-loss data to identify the causal relationship between yield and problematic tools (levels) across different process stages (factors). The recommended process does not directly involve the decision maker’s preference anywhere, which has both advantages and disadvantages. They recommend that the decision maker should at all times evaluate the results by reviewing the identified yield-loss data and key processes to ensure nothing is missing.

Hessinger et al.^7^ suggests methods to select which tools to use for analyzing the source of defects based on inspection tool sensitivity. The methods suggested revolve around yield loss. It also suggests methods to improve inspection effectiveness via defect type filtering and classification. However, the focus of this paper is not to explicitly rank or compare tools.

Madic et al.^8^ propose the use of range of value (ROV) multi-criteria decision making (MCDM) technique to rank cutting fluids. Though ROV is largely unexplored, the authors make the case about its computational simplicity as compared to other MCDM methods. Wang et al. propose an evaluation index for ranking alternative reconfiguration schemes such that it reflects both the advantages and disadvantages of the configurations. The index system is developed using PROMTHEE I and PROMTHEE II and used to rank the various configurations.

Nestic et al.^9^ propose a fuzzy decision-making model to rank manufacturing processes from a quality management perspective in the automotive industry, akin to ranking based on number of defects in this paper. The goal of their model is different however, and is to improve quality management through a fuzzy extension of Elimination and Choice Translating Reality III (ELECTRE III, a family of MCDM). This model assesses and ranks manufacturing sub-processes with respect to key performance indicators.

Khaira and Dwivedi^10^, like in this paper, highlight the importance of identifying the best and worst performing tools. However, their focus is mostly on worst performing, which they refer to as “critical” as they are motivated to assist in maintenance with their models. They propose a two-step decision making for identifying critical section and then critical equipment in that section at an electrode graphite manufacturing plant, a methodology of normalization for the Analytic Hierarchy Process (AHP), and a PROMETHEE based method for validation.

Lyu et al.^11^ propose using the chi-square test of independence, the Apriori algorithm, and the decision tree method identify the sub-process causing defective products and extract rules to identify the lot identification of product defects and their associated manufacturing process parameters. For the analysis they use Internet of Things (IoT) technology to collect manufacturing data.

Elvis et al.^12^ uses MCDM by applying the Delphi method to decide where to place a place a new tool or technology in the automotive manufacturing process.

The aforementioned papers focus on analyzing the source of defects, assist in management, and weed out under performing tools. Our objectives differ from these in that the primary goal of this paper is to rank routes in a system with many tools and hence many potential routes, but when a comparatively much smaller amount of data is available.

We aim to enable an engineer to pick one the potential best routes for testing new recipes. The algorithms we propose do not integrate the decision maker’s preference until the very end, and even then this is optional. Thus, our method is more flexible than many previously developed methods because the decision maker has the option of not tampering with the model at all and utilizing a fully algorithmic ranking produced by our model. Further, the preferences of the decision maker are not taken for the tools being ranked or the steps involved in the process but for the relative importance of the defects with respect to each other. In fact, we incorporate the preference of the decision maker as a weight for a particular defect. This is done separately for each defect and then all the weighted scores are added up.

## Count-based route ranking

As mentioned above, a recipe is a specific set of steps and tool settings required to produce a device. In most cases, each step can be performed by different tools within a tool group. These tools often have slightly different capabilities and performance characteristics. Here, and in subsequent sections, we assume that a number of wafers with known routes have been analyzed. Note that for practical purposes, a route is usually just a small portion of the complete recipe for a wafer.

In this section, we develop ranking algorithms when the metric of interest is a count (i.e., a non-negative integer). Throughout the analysis we assume that a lower count is better. Hence, these techniques make sense when examining the number of defects on a wafer, where a zero count is ideal. For concreteness, we assume that the given data consists of the route taken by a wafer, along with the number of different types of defects incurred per wafer after the wafer has completed the production steps included in the route. We develop two count-based ranking algorithms: (1) the count regression algorithm and (2) the binary ranking algorithm.

It should also be noted that in many processes there are a large number of products with “excessive zeros” from the viewpoint of basic Poisson-based models. This dispersion can be hard to capture using techniques such as adding interaction variables, additional variables and even removing outliers. Count regression methods, as we will see in the next section, allow us to effectively capture this overdispersion and produce better models.

### Count regression algorithm

We regress each defect type separately against each tool according to the regression model described below. Suppose the data set has *m* steps. Let ${n_{j}}$ be the number of tools in step $j \in \{1,2,\ldots ,m\}$, $l \in \{1,2,\ldots ,{n_{j}}\}$ be the *l*th tool of step *j*, and suppose there are *d* different types of defects. For the *l*th tool of the *j*th step, let ${n_{jl}}$ be the number of corresponding sample points from our data set and let $I_{jl}$ be the index set of these sample points (where a “sample point” refers to a route taken by one of wafers and the associated defect data in the data set). For the *s*th sample point in our data, let $y_{is}$ denote the number of defects of type *i* in sample point (or route) *s* of the data set, i.e., $y_{is} \in \mathbb {Z^{+}}$, where $\mathbb {Z^{+}}$ denotes the set of non-negative integers. Let $\mu _{ijl}\in \mathbb {R^{+}}$ denote the average number of defects of type *i* detected on wafers that were processed on tool *l* of step *j*, where $\mathbb {R^{+}}$ denotes the set of non-negative reals. Then,

1 $$\begin{aligned} \mu _{{ijl}} = \frac{\sum _{s\in I_{jl}}y_{is}}{n_{jl}} \quad \forall \ i\in {1,\ldots ,d}; \ j\in {1,\ldots ,m}; \ l\in {1,\ldots ,n_{j}}. \end{aligned}$$

We also define $\mu _{ij}\in \mathbb {R^{+}}$ as the follows:

2 $$\begin{aligned} \mu _{{ij}} = \frac{\sum _{l=1}^{n_{j}}\mu _{ijl}}{n_{j}} \quad \forall \ i\in {1,\ldots ,d}; \ j\in {1,\ldots ,m}. \end{aligned}$$

Let the dummy variables $X_{{jl}}$ be defined as follows:

3 $$\begin{aligned} X_{{jl}} = \left\{ \begin{array}{rl} 1,&{} if \,tool \, l \,is \,used \,in \,step \, j \\ 0,&{} otherwise \end{array} \right. . \end{aligned}$$

Then the count regression model for predicting the average number defects of type *i* associated with step *j* is given by:

4 $$\begin{aligned} g(\mu _{ij}) = \beta _{ij1} + \beta _{ij2}X_{{j2}} + \beta _{ij3}X_{{j3}} + \cdots + \beta _{ij{n_{j}}}X_{{j{n_{j}}}} \end{aligned}$$

where $g(\cdot )$ is the link function for the count regression and the $\beta _{ijl}$’s are the regression coefficients of the dummy variables $X_{{jl}}$. The link function can vary depending on the particular model being used. Each $\beta _{ijl}$ is estimated using its corresponding maximum likelihood estimate $\widehat{\beta }_{ijl}$. The intercept $\beta _{ij1}$ indicates the effect of the first tool of the *j*th step ($j \in \{1,2,\ldots ,m\}$).

The aim of this model is to help us determine the relative effect of the different tools and corresponding steps on defects. To determine the relative contribution of an individual tool within a particular step on the number of defects, we set the dummy variable corresponding to that tool to 1 and dummy variables corresponding to all other tools in that step to 0. Thus, if $X_{j\tilde{l}}=1$ for some tool $\tilde{l}$ of step *j* and $X_{j{l}}=0 \ \forall \ l \ne \tilde{l}$ then Eq. (4) reduces to:

5 $$\begin{aligned} g(\mu _{{ij\tilde{l}}}) = \beta _{ij1} + \beta _{ij\tilde{l}}. \end{aligned}$$

**Figure 1:**
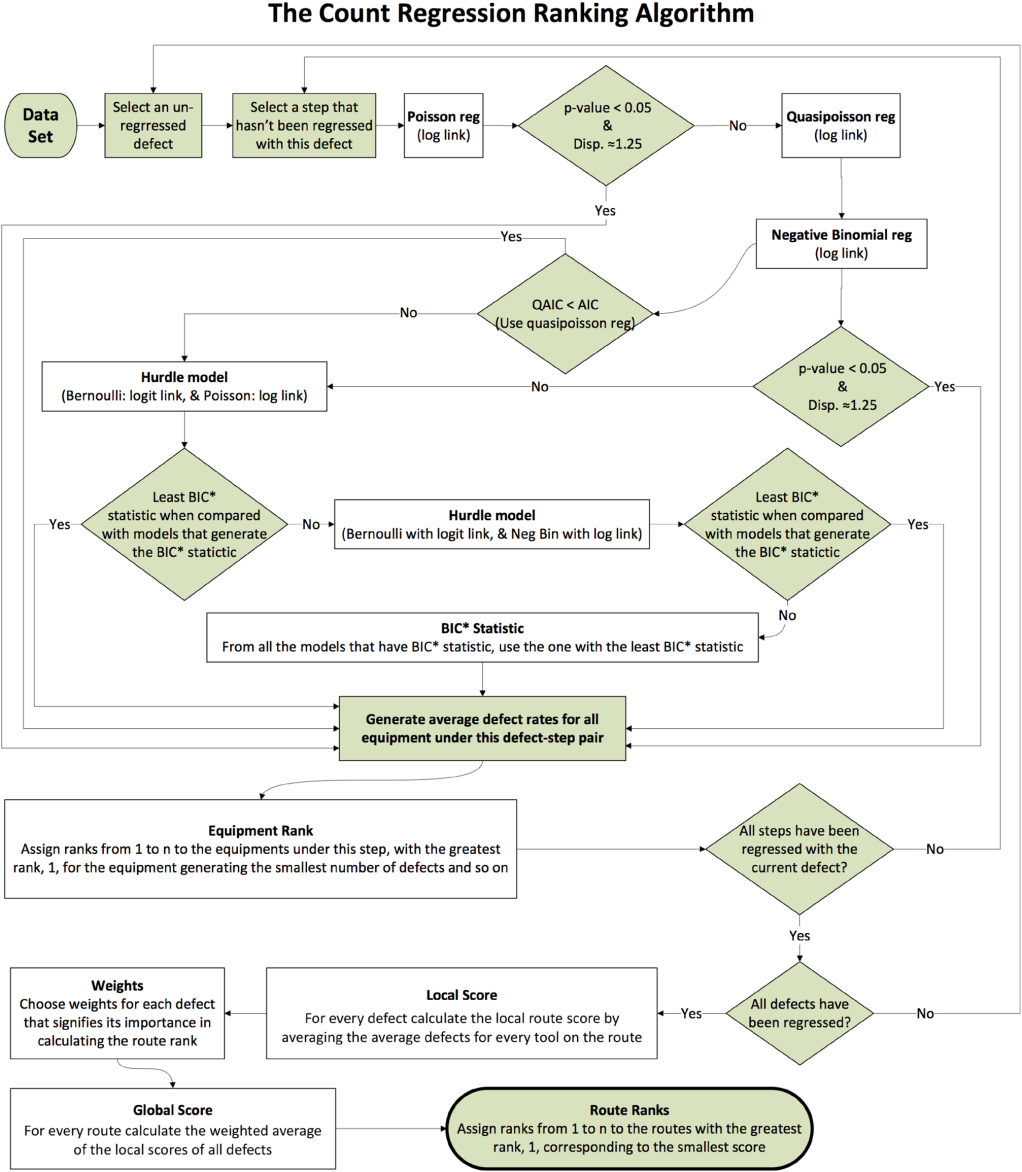
Procedure for ranking routes based on defect count data using count regression.

Having described how to interpret our model, we now proceed to development of the algorithm. The first step is to find a regression model that best describes the defect count data. The algorithm developed to achieve this (represented in Fig. 1) begins with a Poisson regression, which models the logarithm of the expected value of the counts. A general Poisson regression model with a log link is:

6 $$\begin{aligned} ln(\mu _{ij}) = \beta _{ij1} + \beta _{ij2}X_{{j2}} + \beta _{ij3}X_{{j3}} + \cdots + \beta _{ij{n_{j}}}X_{{j{n_{j}}}}. \end{aligned}$$

Again using (5), for a particular tool *l* we have:

7 $$\begin{aligned} ln(\mu _{{ij{l}}}) = \beta _{ij1} + \beta _{ij{l}}. \end{aligned}$$

Thus, the marginal probability of incurring *y* defects of type *i* as an effect of tool *l* of step *j* (note: this does not mean as a direct result of tool *l*) can be determined using the rate $\mu _{ij{l}}$ obtained above via a Poisson probability mass function (pmf):

8 $$\begin{aligned} f_{ij{l}}(y) = \left\{ \begin{array}{ccl} \frac{e^{-\mu _{ij{l}}}\mu _{ij{l}}^{y}}{ y!},&{}&{} y\in \mathbb {Z^{+}} \\ 0,&{}&{} \textrm{otherwise} \end{array} \right. . \end{aligned}$$

In this model we need to check for overdispersion in the defect data because the mean and variance of a count model with a Poisson link should be the same. We estimate overdispersion using the sample mean and variance, $\widehat{\mu }$ and $\widehat{\sigma }^2$, respectively, of the entire defect data set. The defect data is categorized as overdispersed if ${\widehat{\sigma }^2} > {\widehat{\mu }}$ or $\frac{\widehat{\sigma }^2}{\widehat{\mu }} > 1$. The latter expression is called the dispersion statistic. The analysis of overdispersion depends on three things: (1) the value of the dispersion statistic, (2) the number of observations in the model, and (3) the structure of the data.

For the size of the data set we worked with ($\approx 1000$ sample points) and based on the recommendations in the literature, slight overdispersion is permissible as long is it does not exceed 1.25^13^.

If the dispersion is larger than 1.25, we start by first modifying the Poisson regression model using a quasi-likelihood adjustment.

If the model is not overdispersed, then the Pearson $\chi ^{2}$ statistic for a sample of size *n* is:

9 $$\begin{aligned} P^{\chi ^{2}}_{ij}= & {} \displaystyle \sum _{l=1}^{n_{j}}\displaystyle \sum _{s\in I_{jl}} \frac{(y_{is}-{\mu }_{ijl})^{2}}{ \widehat{\sigma }^{2}} = \displaystyle \sum _{l=1}^{n_{j}}\displaystyle \sum _{s\in I_{jl}} \frac{(y_{is}-{\mu }_{ijl})^{2}}{ \widehat{\mu }}. \end{aligned}$$

If the data is not overly sparse and the model is correct then $P^{\chi ^{2}}_{ij} \sim \chi ^{2}_{n-n_{p}}$, where $n_{p}$ is the number of parameters being estimated. If $P^{\chi ^{2}}_{ij}$ indicates lack of fit but the estimated number of defects, $\mu _{ijl}$, is sufficiently close to the true defect value, $y_{is,s \in I_{jl}}$, i.e., the model has a low mean squared error or high adjusted *R*-squared value, then the sample variance might not be correctly capturing the true population variance of the data (assuming the data follows a Poisson distribution)^14^. It is also typical in such a case that the model is overdispersed, i.e.:

10 $$\begin{aligned} \widehat{\sigma }^2 > \widehat{\mu } \implies P^{\chi ^{2}}_{ij} = \displaystyle \sum _{l=1}^{n_{j}}\displaystyle \sum _{s\in I_{jl}} \frac{(y_{is}-{\mu }_{ijl})^{2}}{ \widehat{\sigma }^{2}} < \displaystyle \sum _{l=1}^{n_{j}}\displaystyle \sum _{s\in I_{jl}} \frac{(y_{is}-{\mu }_{ijl})^{2}}{ \widehat{\mu }}. \end{aligned}$$

In this case, a reasonable remedy is to assume that the variance is a multiplicative factor of the assumed population variance for a Poisson distribution, i.e., $var(y_{s}) = \phi \cdot \mu$ for some constant $\phi \in \mathbb {R}$. The model with this adjustment is called a quasi-Poisson model and it involves the following small adjustment to the Pearson chi-square statistic:

11 $$\begin{aligned} \tilde{P}^{\chi ^{2}}_{ij} =\displaystyle \sum _{l=1}^{n_{j}}\displaystyle \sum _{s\in I_{jl}}\frac{(y_{is}-{\mu }_{ijl})^{2}}{\phi _{ij} \cdot \widehat{\sigma }^{2}} = \frac{P^{\chi ^{2}}_{ij}}{\phi _{ij}}, \end{aligned}$$

where $\tilde{P}^{\chi ^{2}}_{ij}$ is the modified Pearson chi-square statistic.

Since a $\chi ^{2}_{n-n_{p}}$ random variable has expected value $n-n_{p}$, one simple way to estimate dispersion is to find a $\widehat{\phi }_{ij}$ that makes Pearson chi-square statistic equal to the mean of the distribution it follows. Thus we set:

12 $$\begin{aligned} \tilde{P}^{\chi ^{2}}_{ij} = n-n_{p} \implies \widehat{\phi }_{ij} = \frac{P^{\chi ^{2}}_{ij}}{n-n_{p}}. \end{aligned}$$

Now, in order to see how this adjustment affects the model and the associated estimates, we note that the Poisson distribution belongs to the exponential family of distributions given by:

13 $$\begin{aligned} f(y; \theta , \phi ) = exp \left\{ \frac{y\theta - b(\theta )}{\alpha (\phi )} + C(y; \phi ) \right\} = exp \left\{ y \log (\mu ) - \mu - \log (y!) \right\} , \end{aligned}$$

where $\theta = \log (\mu )$, $\psi =\mu$, $b(\theta ) = \mu$, $\phi = 1$, $\alpha (\phi )=1$ and $C(y; \phi ) = - \log (y!)$. The variance adjustment $\phi$ given by (12) is the dispersion parameter $\phi$ in (13). This variance adjustment consequently modifies (13) to the exponential family $f(y; \theta , \widehat{\phi })$, which may no longer integrate to unity and should be simply considered a useful modification of the likelihood function $l(\cdot ) = log(f(\cdot ))$(see^14^). However, the main question is: how does this transformation affect our parameter estimates in the count regression model given by (4)? The estimates for the original Poisson regression model given by (4) are obtained via the maximum likelihood estimation (MLE) method. Thus, they are called the MLE estimates and it can shown that they are obtained by setting the following partial derivatives to zero for each tool *l* in each step *j* for each defect:

14 $$\begin{aligned} \frac{\partial l({\varvec{\beta _{ijl}}};{\varvec{y}})}{\partial \beta _{ijl}} = \displaystyle \sum _{s=1}^{n}\frac{{\partial \mu _{js} \over \partial \beta _{ijl}}(y_{is}-\mu _{js})}{var(y_{is})} = \displaystyle \sum _{s=1}^{n}\frac{{\partial \mu _{js} \over \partial \beta _{ijl}}(y_{is}-\mu _{js})}{\sigma } \, \end{aligned}$$

where ${\varvec{\beta _{ijl}}}$ and ${\varvec{y_{ijl}}}$ are vectors of the parameter set and all the data points, respectively. Therefore, when only the variance changes with an adjustment factor of $\widehat{\phi }$ given by (12), the MLE estimates above are just scaled by a factor of $\widehat{\phi }$ as follows:

15 $$\begin{aligned} \frac{\partial l({\varvec{\beta _{ijl}}};{\varvec{y}})}{\partial \beta _{ijl}} = \displaystyle \sum _{s=1}^{n}\frac{{\partial \mu _{js} \over \partial \beta _{ijl}}(y_{is}-\mu _{js})}{\widehat{\phi } \cdot \sigma } = \frac{1}{\widehat{\phi }}\displaystyle \sum _{s=1}^{n}\frac{{ \frac{\partial \mu _{js}}{\partial \beta _{ijl}} }(y_{is}-\mu _{js})}{\sigma }. \end{aligned}$$

Thus, the MLE estimates for $\widehat{\beta }_{ijl}$ remain unchanged.

This implies that the parameter estimates of the count regression model (4) remain unchanged but the likelihood test statistics and differences in deviation (such as the Pearson chi-squared test statistic) must be divided by $\widehat{\phi }$ before evaluating the goodness of fit using “an appropriate $\chi ^{2}$ distribution” (see^14^). After the quasipoisson adjustment, we build a negative binomial regression model that can account for even larger overdispersion in count data. Between the quasipoisson adjusted and negative binomial regression models, we choose the better model using the information criteria statistics described below.

For the negative binomial regression we use the most popular parameterization which is a Poisson-gamma mixture model that leads to a variance function which is quadratic in the mean. This is known as the NB-2 model, the derivation and motivation of which are outlined in^15^. The NB-2 pmf with mean $\mu$ and variance $\mu + \alpha \mu ^{2}$ is:

16 $$\begin{aligned} f_{ij}(y|\mu ,\alpha ) = \frac{\Gamma (y+\alpha ^{-1})}{\Gamma (y+1)\Gamma (\alpha ^{-1})} \left( \frac{\alpha ^{-1}}{\alpha ^{-1} + \mu } \right) ^{\alpha ^{-1}} \left( \frac{\mu }{\alpha ^{-1} + \mu }\right) ^{y}. \end{aligned}$$

The corresponding count regression model with a $\log$ link is the same as in (6). However, this model also may not turn out to be a good fit (i.e., it may have *p* value $\ge 0.05$). One of the reasons for a bad fit may be that the negative binomial (NB-2) overdispersion (which also implies Poisson overdispersion) of the estimated variance of the predicted defect rates (while modeling each individual defect of type *i* and each individual step *j*) is greater than $\mu + \alpha \mu ^{2}$^13,15^. If the model variations we have considered so far fail to provide a good fit to the data, we may need to consider another issue: overdispersion may be due to excess zeros. As such, we proceed to work with hurdle models as a final step. The hurdle model is a two part model. The first part is a Bernoulli process which models the probability of getting a zero defects versus getting a positive number of defects (irrespective of the magnitude of this number). This can be achieved using a probit, logit or complementary log-log model. The second part involves modeling count data as a zero-truncated Poisson, geometric or negative binomial model. In our framework we used a probit link for the Bernoulli model, and a Poisson or negative binomial distribution for the positive counts. Thus we have two types of hurdle models, one with a Bernoulli hurdle and a Poisson count process, and the other with a Bernoulli hurdle and a negative binomial count process. For the Poisson distribution, given by (8), the probability of a zero count ($e^{-\mu _{ijl}}$) is subtracted from one and the remaining probabilities are rescaled using this difference. So a zero-truncated Poisson (ZTP) distribution has the pmf:

17 $$\begin{aligned} f_{ijl}(y) = \left\{ \begin{array}{ccl} \frac{e^{-\mu _{ijl}}\mu _{ijl}^{y} }{ y! \cdot (1-e^{-\mu _{ijl}})},&{}&{} y \in {\mathbb {N}} \\ 0,&{}&{} \textrm{otherwise} \end{array} \right. . \end{aligned}$$

A similar process is followed for a zero-truncated negative binomial count process. We chose hurdle models over other models such as pure ZTP, a zero-inflated Poisson, or a negative binomial process (ZIP or ZINB). The pure ZTP model is not useful because it completely disregards zero counts. However, zero counts are important to us because a high-zero count indicates a better process. Like the hurdle models, ZIP and ZINB are two-part models consisting of both a Bernoulli process and count process. However, unlike hurdle models, these models assume that the zero counts result from both the binary and the count process. Hurdle models, on the other hand, separate the modeling of zeros from that of counts because it assumes that only one process generates zeros. Hence, we prefer hurdle models and do not pursue the ZTP, ZIP and ZINB models. When determining model fit, hurdle models can be compared with each other and the other models in this section using the Alkaike Information Criterion (AIC) statistic, if the sample size, *n*, is less than 8. Otherwise, the Bayesian Information Criterion (BIC) is recommended. This is because the BIC imposes a stronger penalty on model complexity than the AIC for $n\ge 8$, i.e., when the sample size is large^16^. The AIC and BIC are defined as follows:

18 $$\begin{aligned} \text {AIC}&= -2l(\widehat{{\varvec{\theta }}}) + 2n_{p}, \end{aligned}$$

19 $$\begin{aligned} \text {BIC}&= -2l(\widehat{{\varvec{\theta }}}) + n_{p}\log n, \end{aligned}$$

where $n_{p}$ is the number of model parameters, $\widehat{{\varvec{\theta }}}$ is a vector of MLE parameter estimates obtained by maximizing $l(\widehat{{\varvec{\theta }}})$, which is the log-likelihood. Thus, the AIC is a conservative statistic for measuring the model fit, as quantified by $l(\widehat{{\varvec{\theta }}})$, and model complexity, as quantified by *s*. It should be noted that the quasipoisson model does not generate the AIC statistic because it is not derived using the MLE method. Rather, the quasi-likelihood correction (see (11)–(15)) to the AIC model-selection criterion is given by the quasi-AIC (QAIC):

20 $$\begin{aligned} \text {QAIC} = -2\frac{l(\widehat{{\varvec{\theta }}})}{\widehat{\phi }} + 2n_{p}, \end{aligned}$$

where $\widehat{\phi }$ is the estimated dispersion parameter for the quasi-likelihood. However, using QAIC is useful only when all the models being compared to the quasipoisson model use a common value of $\widehat{\phi }$. For simplicity, we continue to use the *p* value as a metric for determining the significance of the quasipoisson model. The procedure for conducting a count regression analysis for defect count data is shown in Fig. 1. More detailed explanations of each of these models can be found in Hilbe^13^.

We made some assumptions in the algorithm to avoid complexity in model selection:

- If the *p* value was greater than or equal to 0.05 an alternate model was used even when the dispersion was approximately 1.25.
- If the dispersion was not approximately 1.25, an alternate model was used even when the *p* value was $< 0.05.$
- To compare models that do not generate a *p* value, if $n<8$ the AIC is used, otherwise the BIC is used. For example, in our procedure we use these criteria to compare hurdle models with Poisson and NB-2 count models.

Once we identify the count regression model that is most appropriate, we obtain the coefficients describing the effect of the various tools at every step from the following logistic regression equation:

21 $$\begin{aligned} ln(y_{ij}) = \beta _{1} + \beta _{i,2}X_{{j,2}} + \beta _{i,3}X_{{j,3}} + \cdots + \beta _{i,{n_{j}}}X_{{j,{n_{j}}}}. \end{aligned}$$

After determining the coefficients, we transform the equation as follows to obtain the average defect rates for the various tools:

22 $$\begin{aligned} y_{ij} = e^{\beta _{1} + \beta _{i,2}X_{{j,2}} + \beta _{i,3}X_{{j,3}} + \cdots + \beta _{i,{n_{j}}}X_{{j,{n_{j}}}}}. \end{aligned}$$

Thus, average number of defects of type *i* for tool *l* of step *j* is given by:

23 $$\begin{aligned} y_{ijl} = \left\{ \begin{array}{ccl} e^{\beta _{1}},&{}&{} for \,tool \,l=1\, of \,the \,j\text {th}\,step \\ e^{\beta _{1}+\beta _{il}},&{}&{} for \,all \,other \,tools \,(l \ne 1) \,of \,the \, j\text {th} \,step. \end{array} \right. \end{aligned}$$

For hurdle models we have two sets of coefficients for each tool: (1) the binary model coefficients (Bernoulli with a logistic link represented by (24)), and (2) the zero-truncated count model (Poisson or negative binomial with a logistic link represented by (21)). In (24) below, $\frac{p_{{ij}}}{1 - p_{{ij}}}$ is the odds ratio of incurring defect *i* when a tool of the *j*th step is present in the route:

24 $$\begin{aligned} log \left( \frac{p_{{ij}}}{{1 - p_{{ij}}}} \right) = \alpha _{1} + \alpha _{i,2}X_{{j,2}} + \alpha _{i,3}X_{{j,3}} + \cdots + \alpha _{i,{n_{j}}}X_{{j,{n_{j}}}}. \end{aligned}$$

After determining the $\alpha$ coefficients we transform (24) as follows to obtain the average defect rates for various tools:

25 $$\begin{aligned} \frac{p_{{ij}}}{{1 - p_{{ij}}}} = e^{\alpha _{1} + \alpha _{i,2}X_{{j,2}} + \alpha _{i,3}X_{{j,3}} + \cdots + \alpha _{i,{n_{j}}}X_{{j,{n_{j}}}}}. \end{aligned}$$

Thus, the odds ratio of defects of type *i* for tool *l* of step *j* is:

26 $$\begin{aligned} \frac{p_{{ijl}}}{{1 - p_{{ijl}}}} = \left\{ \begin{array}{ccl} e^{\alpha _{1}},&{}&{} for \,tool \,l=1 \,of \,the \,j\text {th} \,step \\ e^{\alpha _{1}+\alpha _{il}},&{}&{} for \,other \,tools \,(l \ne 1) \,of \,the \,j\text {th} \,step. \end{array} \right. \end{aligned}$$

Finally, the probability of incurring a defect *i* if tool *l* is used in the *j*th step is:

27 $$\begin{aligned} p_{{ijl}} = \left\{ \begin{array}{ccl} \frac{e^{\alpha _{1}}}{ 1+e^{\alpha _{1}} },&{}&{} for \,tool \,l=1 \,of \,the \,j\text {th} \,step \\ \frac{e^{\alpha _{1}+\alpha _{il}} }{ e^{\alpha _{1}+\alpha _{il}}},&{}&{} for \,other \,tools \,(l \ne 1) \,of \,the \,j\text {th} \,step. \end{array} \right. \end{aligned}$$

We can then use the law of iterated expectation to obtain the expected number of defects of type *i* generated by the *l*th tool representing the *j*th step from the zero-truncated count regression coefficients generated using (23), and the Bernoulli logistic regression coefficients from (27). The expected number of defects of type *i* generated by the *l*th tool at the *j*th step is the sum of the probability of incurring a positive number of defects multiplied by the average number of positive defects generated by the corresponding tool, and the probability of incurring no defects multiplied by 0. In summary, we have

28 $$\begin{aligned} \begin{array}{rl} E[y_{ijl}] &{}= p_{{ijl}} \cdot y_{ijl} + (1-p_{{ijl}}) \cdot 0\\ &{}= p_{{ijl}} \cdot y_{ijl}\\ \end{array}, \end{aligned}$$

29 $$\begin{aligned} \Rightarrow E[y_{ijl}] = \left\{ \begin{array}{ccl} p_{{ij,1}} \cdot e^{\beta _{1}},&{}&{} for\, tool \,l=1\, of \,the \, j\text {th} \,step\, of\, the\, routes \\ p_{{ijl}} \cdot e^{\beta _{1}+\beta _{il}},&{}&{} for \,other\, tools \,(l\, \ne 1) \,of\, the \, j\text {th}\, step\, of \,the \,routes \end{array} \right. . \end{aligned}$$

Once we obtain the expected number of defects produced by each tool for every step-defect combination, we proceed to ranking routes using the algorithm described in “Global route ranking”.

### Binary rank algorithm

In this subsection, we consider an alternative way to perform the local scoring of tools to produce a rank for each unique tool at each step. In this framework, the metric is whether a tool produces defects or not. Hence, the precise number of defects is immaterial but the probability of a route causing a wafer it processes to have a defect of a particular type is important.

Instead of the complex regression algorithm to obtain the expected number of defects produced by each tool under each every step-defect combination outlined in previous sections, we develop a simpler algorithm. We calculate the probability $q_{ijl}$, which here has a different definition from $p_{ijl}$ described in the previous section on the count-regression algorithm. It is the probability of incurring zero defects of type *i* if tool *l* is used in step *j*. We calculate this quantity below:

30 $$\begin{aligned} q_{ijl} = \frac{\sum _{s\in I_{jl}} (1-y_{is})^+}{n_{jl}} \quad \forall \ i\in {1,\ldots ,d}; \ j\in {1,\ldots ,m}; \ l\in {1,\ldots ,n_{j}}. \end{aligned}$$

The rest of the terms $s,I_{jl},\mathbb {Z^{+}},y_{is},n_{jl},d,m$ and $n_{j}$ have the same meanings in the previous section.

**Figure 2:**
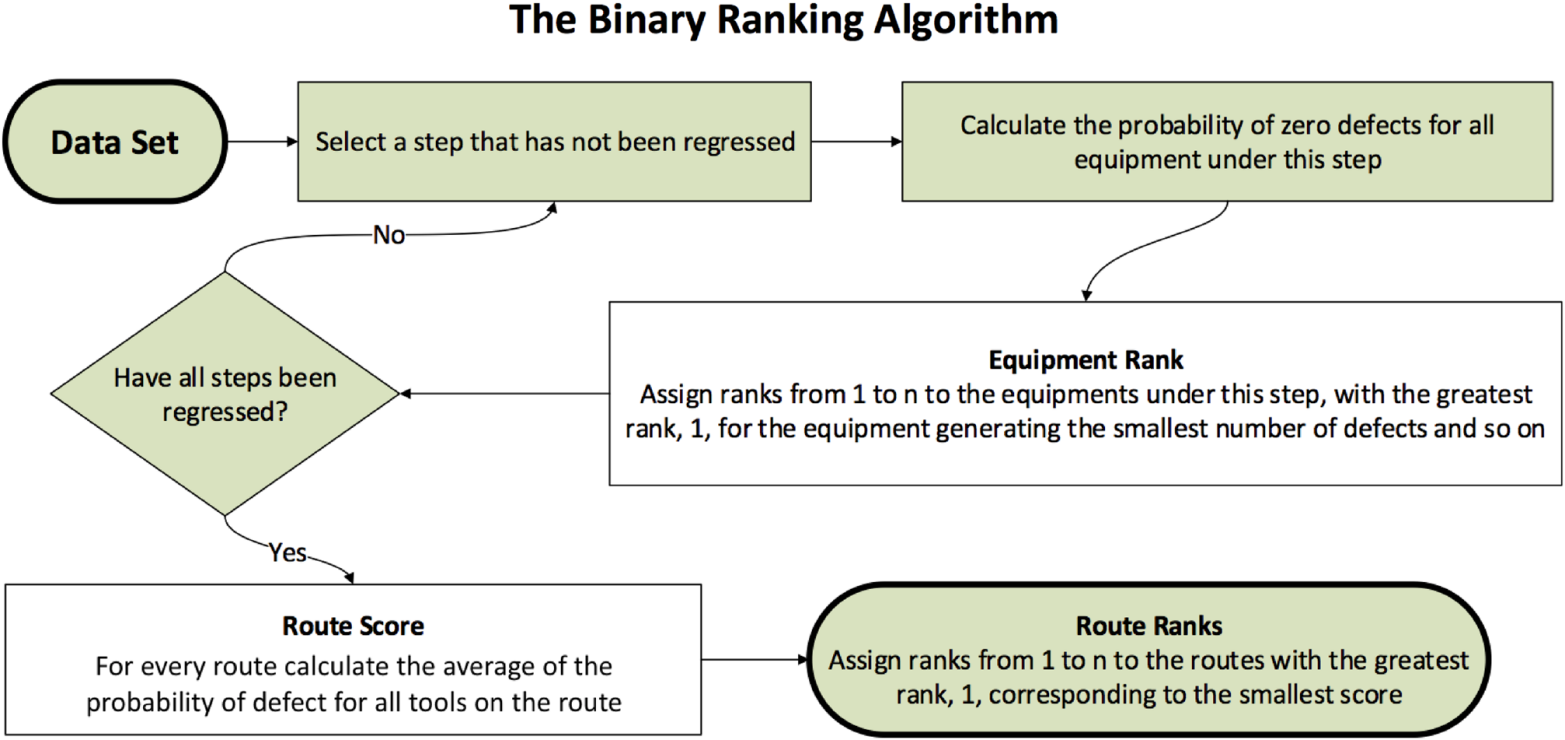
The binary ranking algorithm for ranking routes using defect count data.

Finally, just like in the precious section, we use these probabilities (let’s call them tool scores for uniformity across the two algorithms) obtained for each step and and calculate the route ranks using the procedure described in “Global route ranking”. The flowchart in Fig. 2 depicts the algorithm.

### Global route ranking

For each defect and each route we first produce a *local score* by further averaging the average defects (in the case of count regression ranking), or average probability of defects (in the case of binary ranking), for every tool on the given route and the given defect. We call this the local score because it is specific to a defect type. Thus, for each defect *i*, we obtain a score $s_{it}$ of the *R* routes, where $i \in \{1,2,\ldots ,d\}$ and $t \in \{1,2,\ldots ,R\}$.

Finally, we calculate the *global rank* $r_{t}, t \in \{1,2,\ldots ,R\}$, of the routes by taking a weighted average of the local scores for the routes (let’s call this average the *global score*) and ranking them from 1 through *N* ($N\le R$ because there is a possibility of tied ranks for some routes). Here rank 1 corresponds to the route(s) associated with the smallest number (or probability) of average defects and rank *N* corresponds to the route(s) associated with the maximum number (or probability) of average defects. Using the local scores in place of local ranks helps drive more uniqueness in global route ranks as simply ranking the sum of the local ranks may cause many more routes to have the same rank, whereas summing local scores allows more uniqueness in the global scores, and thus, in global ranks. Selecting the weights $w_{i}$, $i \in \{1,2,\ldots ,d\}$ for each defect in order to calculate the weighted average is up to the stake holders and decision makers. Our formulation is encapsulated in the equations below:

31 $$\begin{aligned} s_{it}&= \displaystyle \sum _{j=1}^{m}\displaystyle \sum _{l=1}^{{n_{j}}}s_{ijlt}&\quad \forall \ \text {defects } i = 1,\ldots ,d; \end{aligned}$$

32 $$\begin{aligned} r_{t}&= Rank \left( \frac{1}{d} \displaystyle \sum _{i=1}^{d} w_{i} \cdot s_{it} \right) = Rank \left( \frac{1}{d} \displaystyle \sum _{i=1}^{d} w_{i} \displaystyle \sum _{j=1}^{m} \displaystyle \sum _{l=1}^{{n_{j}}} s_{ijlt} \right)&\quad \forall \ \text {routes } t = 1,\ldots ,m; \end{aligned}$$

where $s_{ijlt}$ is the local score of the *l*th tool at the *j*th step in the *t*th route obtained for the *i*th defect. An example of weighted global route ranks using the count regression and binary rankings method are shown in Tables 4 and 5, respectively.

## Computational examples

The semiconductor data set we worked with had four defect types. Wafers on which no defects were observed were also recorded. The routes in the data set all had eleven steps, and each step had its own set of tools. Steps 1 through 11 had 5, 14, 5, 14, 11, 5, 11, 9, 4, 10 and 13 distinct tools, respectively. All possible combinations of these distinct tools under each of the eleven steps generated approximately $1.4\times 10^{10}$ possible routes, while only 652 of these were actually represented in our data set containing 2 months of fab data.

Following the steps of count regression as outlined in the flowchart in Fig. 1, we compute metrics for the various models used, including the dispersion, *p* value and AIC statistics. An example can be seen in Table 1. Once the best model is obtained, the tools under each step are scored separately using the best count regression algorithm for each defect-step combination (cf. Table 2 for sample results). We also re-derive all the tool scores using the binary ranking algorithm. A sample of these results are in Table 2. We then obtain the local and global route scores and ranks as shown in Tables 3, 4 and 5. More detailed computational results on this data set are available in^17^. Further, the ranks obtained by the two different algorithms are compared using rank correlations which is described and discussed in the next section.

**Table 1:** Sample output obtained using the count regression algorithm.

Defect	Step	Regression type	*p* value	Dispersion	AIC	Best fit
def1	Step1	Poisson	0	3.7297	6601.7436	No**
def1	Step1	Quasipoisson	0	3.7297	1e+07	No**
def1	Step1	Negative binomial	0.0243	1.0894	5058.6243	Yes
def1	Step1	Hurdle-binomial, Poisson	NA in R*	NA in R*	6368.3306	No
def1	Step1	Hurdle-binomial, negative binomial	NA in R*	NA in R*	5066.7132	No
def1	Step2	Poisson	0	3.6935	6594.0904	No**
def1	Step2	Quasipoisson	0	3.6935	1e+07	No**
def1	Step2	Negative binomial	0.0164	1.0976	5100.3177	Yes
def2	Step3	Poisson	0	2.5180	3487.7709	No**
def2	Step3	Quasipoisson	0	2.5180	1e+07	No**
def2	Step3	Negative binomial	0.9999	0.7743	2466.1977	No
def2	Step3	Hurdle-binomial, Poisson	NA in R*	NA in R*	3040.5102	No
def2	Step3	Hurdle-binomial, negative Binomial	NA in R*	NA in R*	2439.6879	Yes
def2	Step4	Poisson	0	2.5110	3520.4343	No**
def2	Step4	Quasipoisson	0	2.5110	1e+07	No**
def3	Step3	Poisson	0	1.6522	2192.9523	No**
def3	Step3	Quasipoisson	0	1.6522	1e+07	No**
def3	Step3	Negative binomial	1	0.4831	1606.0632	No**
def3	Step3	Hurdle-binomial, Poisson	NA in R*	NA in R*	1708.7439	No
def3	Step3	Hurdle-binomial, negative binomial	NA in R*	NA in R*	1636.4713	Yes

This output displays the count regression models that were a best fit to the different *step-defect* data slices.

$^{*}$This value could not be extracted for the model using R.

$$^{**}$$This model was not considered as the best fit in spite of producing a significant *p*-value $$> \alpha (= 0.5)$$, the dispersion was $$\not \approx 1.25$$ and because another model (Hurdle-Binomial, Negative Binomial) yielded a lower AIC statistic. This depicts the robustness of our algorithm.

**Table 2:** A sample of the ranks of different tools for the various defects using the count regression ranking algorithm.

Defect	Step1	Equipment	Average defects per tool per step via count regression
def1	Step1	EQP_31	48.79
def1	Step1	EQP_32	62.26
def1	Step1	EQP_35	45.84
def1	Step1	EQP_36	63.71
$\cdot$	$\cdot$	$\cdot$	$\cdot$
$\cdot$	$\cdot$	$\cdot$	$\cdot$
$\cdot$	$\cdot$	$\cdot$	$\cdot$
def1	Step11	EQP_60	48.23
def1	Step11	EQP_61	79.25
def1	Step11	EQP_62	77.98
def1	Step11	EQP_50	56.00

**Table 3:** Sample local route ranks obtained for defect 1 using the count regression algorithm.

Route			Count regression ranks				
Step 1	Step 2	Step 3	Step 1	Step 2	Step 3	Local score	Local rank
EQP_35	EQP_16	EQP_49	45.84	1.61	4.65	17.37	1
EQP_38	EQP_16	EQP_48	61.47	1.61	3.50	22.20	3
EQP_32	EQP_10	EQP_48	62.26	2.00	3.50	22.59	4
EQP_31	EQP_16	EQP_49	48.79	1.61	4.65	18.35	2

For brevity, we are restricting a route to only three steps in this example.

**Table 4:** This table is shows sample weighted global ranks for routes obtained using the count regression ranking algorithm.

Route			Defect-1 ranks			Defect-2 ranks			Local scores		Route statistics	
Step1	Step2	Step3	Step1	Step2	Step3	Step1	Step2	Step3	Def 1	Def 2	Weighted global scores	Ranks
EQP35	EQP16	EQP49	45.84	1.61	4.65	0.46	0.50	0.53	17.37	0.50	8.93	1
EQP38	EQP16	EQP48	61.47	1.61	3.50	0.40	0.50	0.71	22.20	0.54	11.37	3
EQP32	EQP10	EQP48	62.26	2.00	3.50	0.42	2.00	0.71	22.59	1.04	11.82	4
EQP31	EQP16	EQP49	48.79	1.61	4.65	0.47	0.50	0.53	18.35	0.50	9.43	2

For brevity, we are restricting a route to only three steps in this example and only consider two defects (defects 1 and 2). Global score is calculated using equal weights of 1.

**Table 5:** This table is shows sample ranks for routes obtained using the binary ranking algorithm.

Route			Defect-1 ranks			Defect-2 ranks			Local scores		Route statistics	
Step1	Step2	Step3	Step1	Step2	Step3	Step1	Step2	Step3	Def 1	Def 2	Weighted global scores	Ranks
EQP35	EQP16	EQP49	0.126	0.050	0.094	0.660	0.583	0.599	0.090	0.614	0.352	2
EQP38	EQP16	EQP48	0.109	0.050	0.109	0.661	0.583	0.632	0.089	0.625	0.357	3
EQP32	EQP10	EQP48	0.088	0.171	0.109	0.588	0.585	0.632	0.123	0.602	0.362	4
EQP31	EQP16	EQP49	0.138	0.050	0.094	0.581	0.583	0.599	0.094	0.588	0.341	1

For brevity, we are restricting a route to only three steps in this example and only consider two defects (defects 1 and 2). Global score is calculated using equal weights of 1.

### Rank correlations

In this section, we review common methods for comparing two rankings for a set of objects. We then use these metrics to compare different route ranking approaches. Suppose there are *t* items, which are assigned rankings in the set $\{1, \ldots , t\}$. For a fixed item *i*, let $\mu (i)$ and $\nu (i)$ be the ranks obtained by two different methods.

#### Spearman metric

We first consider the Spearman distance. In form, it is similar to a Euclidean distance and is given by:

33 $$\begin{aligned} d_{S}(\mu ,\nu ) = \frac{1}{2}\displaystyle \sum _{i=1}^{t}(\mu (i) - \nu (i))^{2} \end{aligned}$$

Note that it is not a proper distance metric because it does not satisfy the triangle inequality. This leads us to the Spearman correlation given by:

34 $$\begin{aligned} \alpha _{S}(\mu ,\nu ) = \frac{2d_{S}(\mu ,\nu )}{M_{S}}, \end{aligned}$$

where

35 $$\begin{aligned} c_{S}&= \frac{t(t^{2}-1)}{12}, \end{aligned}$$

36 $$\begin{aligned} M_{S}&= 2c_{S} . \end{aligned}$$

$c_{S}$ is known as the average Spearman distance and $M_{S}$ as the maximum Spearman distance.

#### Kendall metric

The Kendall distance distance counts the number of *discordant* pairs, i.e., the number of times the ranks of two items are reversed. It is given by:

37 $$\begin{aligned} d_{K}(\mu ,\nu ) = \displaystyle \sum _{i<j}\left[ 1-sgn(\mu (j) - \mu (i)) \cdot sgn(\nu (j) - \nu (i))\right] . \end{aligned}$$

The Kendall correlation is given by:

38 $$\begin{aligned} \alpha _{K}(\mu ,\nu ) = \frac{2d_{K}(\mu ,\nu )}{M_{K}}, \end{aligned}$$

where

39 $$\begin{aligned} c_{K}&= \frac{t(t-1)}{2}, \end{aligned}$$

40 $$\begin{aligned} M_{K}&= 2c_{K} . \end{aligned}$$

$c_{K}$ is the average Kendall distance and $M_{K}$ is the maximum Kendall distance.

The Spearman and Kendall rank correlations between the two ranking methods discussed in this paper are listed in Table 6. We can see that both correlations are statistically significant and very low, Spearman rank at 27.2% and Kendall tau at 18.49%. This implies that ranking by mere probability (binary ranking) is very different from ranking by taking both probability and magnitude of a defect (count regression based ranking). Since the ground truth ranking is not known, it’s hard to say which ranking is better but we recommend that the stakeholder decide what they are interested in. We cover the different use cases of interest for stakeholders in the conclusion.

**Table 6:** Spearman and Kendall correlations between the different ranking methodologies when applied over the dummy data.

Correlation method	Rank correlation (%)	p value
Spearman rank $\alpha _{S}(\mu ,\nu )$	27.20	1.9e−36
Kendall tau $\alpha _{K}(\mu ,\nu )$	18.49	2.7e−36

## Conclusion

Our approach and modeling has several limitations which also create ample opportunities for future work. First and foremost, we do not compare our algorithms with past algorithms in the literature review for the following reasons. Methods such as AHP and PROMTHEE use a lot of qualitative features which we did not have access to in our data set. Hence, we could not reproduce these results. Many authors have also not made their algorithms open source, making comparisons difficult due to lack of reproducibility. In the literature as well authors are seen comparing their algorithms to their own past work but not with other models. Further, pretty much all past work focuses explicitly on ranking only tools and their goal is to identify the worst performing tools. We have made our code public on github (removed link for review process to preserve anonymity of authors, will add back after review process) in case authors going forward want to benchmark their algorithms against ours.

Secondly, the models we tested did not account for the statistical interaction between different tools. The reason for this was an explosion in the number of parameters and compute limitations since we developed and tested in R. However, this should be possible to test in more advanced statistical softwares and with ample compute power. There is definitely interaction between tools, thus, their could be pairs of tools that are superior. One way to reduce the number of parameters would be to only consider consecutive pairs of tools.

Thirdly, we do not compare the count-based and binary algorithms directly because they serve different purposes. While the count regression algorithm is more complex than the binary ranking algorithm, for the data set we tested, the algorithm can produce rankings within minutes and thus it should still be suitable for use in actual fab environments. In fact, the computation time for our data set was roughly the same for both ranking methods. Thus, the choice of method depends on the particular use case. If the decision maker’s goal is to produce the lowest total number of defects, then the count regression algorithm is more appropriate. However, if the goal is to produce the highest number of defect-free wafers, then the binary algorithm is preferable.

As for future research, an important step is to test the algorithms on larger data sets. With the advances in machine learning, as and when more data is available, more complex ranking algorithms like RankNet, LambdaRank or LambdaMART could be also employed^18–20^. When using regression or machine learning models, explainability models like Local Interpretable Model-Agnostic Explanations (LIME)^21^ and SHapley Additive exPlanations (SHAP)^22^ can be used to understand which tools are driving the rank of a route up or down. Counterfactual analysis using these explanation methods could also be useful in extending the purpose of this work to recognize which tools could be worked on so their throughput is of higher quality. Furthermore, it would be useful to create a methodology to do rolling, or online, updates to rankings perhaps using some sort of smoothing or Bayesian approach.

## Data Availability

The datasets generated during and/or analysed during the current study are available from the corresponding author on reasonable request.
